# Molecular Characterization of *Cronobacter sakazakii* Strains Isolated from Powdered Milk

**DOI:** 10.3390/foods10010020

**Published:** 2020-12-23

**Authors:** Ondrej Holý, Julio Parra-Flores, Sarah Lepuschitz, María Paula Alarcón-Lavín, Ariadnna Cruz-Córdova, Juan Xicohtencatl-Cortes, Jetsi Mancilla-Rojano, Werner Ruppitsch, Stephen Forsythe

**Affiliations:** 1 Department of Public Health, Palacký University Olomouc, 77515 Olomouc, Czech Republic; 2 Department of Nutrition and Public Health, Universidad del Bío-Bío, Chillán 3800708, Chile; mpalarcon@ubiobio.cl; 3 Austrian Agency for Health and Food Safety, Institute for Medical Microbiology and Hygiene, 1220 Vienna, Austria; sarahlepuschitz@gmail.com (S.L.); werner.ruppitsch@ages.at (W.R.); 4 Intestinal Bacteriology Research Laboratory, Hospital Infantil de México Federico Gómez, Mexico City 06720, Mexico; ariadnnacruz@yahoo.com.mx (A.C.-C.); juanxico@yahoo.com (J.X.-C.); mancillajetsi@gmail.com (J.M.-R.); 5 Biological Sciences Graduate Program, Facultad de Medicina, Posgrado en Ciencias Biológicas, Universidad Nacional Autónoma de México, Mexico City 04510, Mexico; 6 Adams Hill, Keyworth, Nottinghamshire NG12 5GY, UK; steve.forsythe@foodmicrobe.com

**Keywords:** *Cronobacter sakazakii*, whole-genome sequencing, powdered milk, virulence, antibiotic resistance genes, CRISPR-Cas

## Abstract

*Cronobacter* spp. are opportunistic pathogens of the *Enterobacteriaceae* family. The organism causes infections in all age groups, but the most serious cases occur in outbreaks related to neonates with meningitis and necrotizing enterocolitis. The objective was to determine the in silico and in vitro putative virulence factors of six *Cronobacter sakazakii* strains isolated from powdered milk (PM) in the Czech Republic. Strains were identified by MALDI-TOF MS and whole-genome sequencing (WGS). Virulence and resistance genes were detected with the Ridom SeqSphere+ software task template and the Comprehensive Antibiotic Resistance Database (CARD) platform. Adherence and invasion ability were performed using the mouse neuroblastoma (N1E-115 ATCCCRL-2263) cell line. The CRISPR-Cas system was searched with CRISPRCasFinder. Core genome MLST identified four different sequence types (ST1, ST145, ST245, and ST297) in six isolates. Strains 13755-1B and 1847 were able to adhere in 2.2 and 3.2 × 10^6^ CFU/mL, while 0.00073% invasion frequency was detected only in strain 1847. Both strains 13755-1B and 1847 were positive for three (50.0%) and four virulence genes, respectively. The *cpa* gene was not detected. Twenty-eight genes were detected by WGS and grouped as flagellar or outer membrane proteins, chemotaxis, hemolysins, and invasion, plasminogen activator, colonization, transcriptional regulator, and survival in macrophages. The colistin-resistance-encoding *mcr-9.1* and cephalothin-resis-encoding *bla_CSA_* genes and IncFII(pECLA) and IncFIB(pCTU3) plasmids were detected. All strains exhibited CRISPR matrices and four of them two type I-E and I-F matrices. Combined molecular methodologies improve *Cronobacter* spp. decision-making for health authorities to protect the population.

## 1. Introduction

The *Cronobacter* genus was first defined by Iversen et al. [1] and further developed by Iversen et al. [2] and Joseph et al. [3] to include the *C. sakazakii*, *C. malonaticus*, *C. universalis*, *C. turicensis*, *C. muytjensii*, *C. dublinensis*, and *C. condimenti* species [3]. The population groups most affected by *Cronobacter* spp. are newborns and the elderly. Holý et al. [4] isolated *Cronobacter* spp. in 82 of 45,000 analyzed samples and found an incidence (×1000 samples) of 8.7, 8.2, and 4.8 in infants under 1 y of age, children between 1 and 4 y, and adults over 65, respectively. The fatality rates associated with general infection range from 42% to 80% and 15% to 25% for neonatal meningitis and septicemia, respectively [5]. The highest incidence and severity has occurred in infants, and a number of outbreaks in neonatal intensive care units have been reported [5,6]. Clinical symptoms in infants are primarily meningitis, septicemia, or necrotizing enteritis [7,8,9]. Other symptoms can include diarrhea and urinary tract infection.

Infections are often associated with the consumption of reconstituted powdered infant formula (RPIF) caused by either intrinsic contamination of powdered infant formula (PIF) or extrinsic contamination of preparation utensils and equipment [10]. Given that it is widespread, *Cronobacter* spp. can be isolated from PIF, powdered milk, infant cereals and other products, and water [11,12]. Controlling the organism during PIF production is important because *Cronobacter* spp. strains have been isolated up to 24 months after the PIF was packaged [13]. Studies of *Cronobacter* spp. incidence in PIF have shown an incidence ranging from 3% to 30% [14,15,16,17].

The antibiotic resistance profile of *Cronobacter* is important because the population group consuming PIF and infant products is immunologically vulnerable. Molloy et al. [18] reported that 51% of 33 *C. sakazakii* isolated strains were resistant to cephalothin. Carvalho et al. [19] indicated high resistance to cefazolin (94.4%) and low resistance to amoxicillin (9.45%), cefpodoxime (5.55%), streptomycin (1.35%), and trimethoprim/sulfamethoxazole (1.35%). An 80% resistance to cephalothin was reported in another study [20]. Fei et al. [21] found varying degrees of resistance to cefazolin and 100% resistance to amoxicillin-clavulanate, ampicillin, and cefazolin in 70 strains of *C. sakazakii* isolated from PIF and corresponding processing environments.

Many virulence traits have been identified in *Cronobacter* spp. [22,23], including the association with inositol fermentation [24], invasion and adherence in cellular lines such as HEp-2 and CaCo, the presence of endotoxins [25], detection of virulence genes *cpa*, *hly*, and *sip* [22,26], flagella [27], and the *ompA* and *ompX* genes [28]. In addition, there are other factors such as the use of sialic acid and the presence of a capsule [29]. Strains related to severe cases of meningitis are also associated with clonal complex 4 (CC4) [30]; therefore, it is suggested that not all *C. sakazakii* strains are equally virulent [6].

Molecular subtyping has long been considered as a useful tool in epidemiological surveillance to establish the similarity of bacteria colonizing a particular ecological habitat. Sub-typing of *Cronobacter* spp. has used pulsed-field gel electrophoresis (PFGE), multilocus sequence typing (MLST), and, recently, typing by CRISPR-Cas characterization systems [31]. This is possible because the matrices that constitute these systems can differ among closely related strains, since the genetic information acquired as a result of different exposures to phages and plasmids leads to variations in the repeated sequences and spacers that form them. Therefore, the CRISPR-cas profiles can show a better resolution compared with MLST and PFGE; for this reason, they are used to differentiate highly related strains such as *Cronobacter* spp. Recently, CRISPR-Cas typing was reported as a valuable tool when developing new methods for the early diagnosis of infectious diseases [32]. Whole-genome sequencing (WGS) is a modern tool for the genomic research of *Cronobacter* spp., and it can be used to expand the original 7-loci MLST scheme to 1836 loci of core genome MLST (cgMLST) [33]. The use of the MLST scheme for clinical, food, and environmental isolates has provided information that is relevant for *Cronobacter* spp. and its sources of isolation to analyze the severity of the clinical manifestation in several age groups [34].

The complete genome studies of pathogenic strains have greatly improved our knowledge about the virulence and antibiotic resistance genes [35]. This knowledge leads to an understanding of how pathogens survive by using various mechanisms to infect and elicit variable host disease responses. The aim of the present study was to perform a molecular characterization of six *C. sakazakii* strains isolated from powdered milk in the Czech Republic between 2010 and 2014 in order to identify their virulence potential and the possible presence of antibiotic resistance genes.

## 2. Materials and Methods

### 2.1. Strains Used in This Study

Strains 1847, 13755-1A, 13755-1B, 12683-2A, 12683-1, and 6227 were used in this study and were isolated from 1450 powdered milk samples produced in the Czech Republic between 2010 and 2014 by three manufacturers, A (n = 1), B (n = 4), and C (n = 1).

### 2.2. Isolation and Primary Species Identification of Cronobacter Sakazakii Isolates

The *C. sakazakii* strains were isolated according to the methodology described by Iversen et al. [36] and conserved at −18 °C. Isolates were cultured on Columbia blood agar plates (bioMérieux, Marcy-l’Étoile, France) overnight at 37 °C to identify them. Primary species identification from single colonies was performed by matrix-assisted laser desorption/ionization time-of-flight mass spectrometry (MALDI-TOF-MS) (Bruker, Billerica, MA, USA) and with the MBT Compass IVD software 4.1.60 (Bruker) described by Lepuschitz et al. [37].

### 2.3. Whole-Genome Sequencing (WGS) Data Analysis

The DNA isolation, quantification, and preparation of sequence-ready libraries for whole-genome sequencing (WGS) were as indicated by Lepuschitz et al. [38]. The Sequencing Coverage Calculator (https://www.illumina.com) was used to calculate a desired mean coverage > fold-80.

Raw reads were de novo assembled with the SPAdes version 3.9.0 [39] and processed with the SeqSphere+ software version 5.1.0 (Ridom GmbH, GmbH, Münster, Germany). A gene-by-gene genome-wide comparison was performed for bacterial typing by the MLST+ task template function of SeqSphere+, as previously described [40], and the core genome multilocus sequence type (cgMLST) gene set was defined according to Lepuschitz et al. [38]. Based on the defined cgMLST scheme, isolates were visualized as a minimum spanning tree (MST) to identify genotypic relationships. Sequences of the seven housekeeping genes of the usual MLST scheme were extracted and queried against the *Cronobacter* MLST database and the sequence types (STs) in silico were determined [6].

### 2.4. O-Serotype Determination Analysis

The gene clusters of the *gnd* and *galF* loci are specific to the O-serotype region. They were identified by analyzing WGS sequences via the BIGSdb tools in the PubMLST database (http://pubmlst.org/cronobacter/) [31].

### 2.5. Adherence Assay

The mouse neuroblastoma (N1E-115 ATCC CRL-2263, Manassas, USA) cell line was used for the adherence assay. The N1E-115 cell line was cultured in Dulbecco’s Modified Eagle Medium (DMEM) with 4.5 g/L glucose (GIBCO, MA, USA). In addition, it was supplemented with 7% fetal bovine serum (FBS) (GIBCO, MA, USA) and differentiated in DMEM medium to which 2% FBS and 1.25% dimethyl sulfoxide was added for 5 d. The cells (1 × 10^5^ cells/mL) were sown in 24-well plates (Corning Life Sciences, NY, USA) and infected at a multiplicity of infection100:1. All the studied isolates were previously cultured in Luria broth (LB). Infection was carried out at 37 °C for 4 h and 5% CO_2_. After incubation, the cells were washed with PBS 1× and the bacteria removed by adding 1 mL 0.1% Triton X-100 (Amresco, OH, USA). Afterward, serial dilutions were plated on LB to determine the colony-forming units (CFU) of bacteria adhering to the N1E-115 cell [22]. This assay was repeated twice and in duplicate; the data were expressed as the means plus the standard deviation of the assay results.

### 2.6. Invasion Assay

The conditioning of the monolayers of cell line N1E-115 and infection time were performed as previously described in Section 2.5. After a 4-h incubation, the infected monolayers were washed with PBS 1× and incubated with 1 mL DMEM with 300 µg/mL lysozyme (Sigma-Aldrich, MI, USA) and 100 µg/mL gentamicin (Sigma-Aldrich, MI, USA) at 37 °C for 2 h and 5% CO_2_. After incubation, cells were washed three times with PBS 1×, detached with 1 mL 0.1% Triton X-100, and plated on LB. Invasion frequencies were calculated as the number of bacteria surviving incubation with gentamicin and lysozyme divided by the total number of bacteria present when this antibiotic is not used (bacterial adherence) [22]. This assay was conducted twice and in duplicate; data were expressed as invasion frequency (%).

### 2.7. In Vitro Virulence Gene Detection

Plasminogen activator (*cpa*), hemolysin (*hly*), siderophore-interacting protein (*sip*), flagellin (*fliC*), autotransporter (aut), and outer membrane protein (*ompA*) genes were detected by polymerase chain reaction (PCR) [23]. Amplified products were stained and visualized on 1.5% agarose gel with a 1.0 mg/mL ethidium bromide solution using a gel imaging system [22,23].

### 2.8. Antibiotic Resistance Profile

The disk diffusion method was used in accordance with the recommendations of EUCAST 2019. Commercial antibiotic disks were used and consisted of ampicillin (10 μg), amikacin (30 μg), levofloxacin (5 μg), cephalothin (30 μg), cefotaxime (30 μg), ceftriaxone (30 μg), chloramphenicol (30 μg), gentamicin (10 μg), netilmicin (30 μg), nitrofurantoin (300 μg), cefepime (30 μg), and sulfamethoxazole trimethoprim (25 μg). The resistance/susceptibility profiles were determined according to the manufacturer’s instructions. The *Escherichia coli* ATCC 25922 strain was used as a control.

### 2.9. In Silico Detection of Virulence and Antibiotic Resistance Genes from Whole Genome Sequencing (WGS) Data

The existence of virulence genes was confirmed by the Task Template function in SeqSphere+ for WGS data included in the virulence factor database (http://www.mgc.ac.cn/VFs/). Thresholds for the target scan procedure were set at ≥ 90% to identify the reference sequence and ≥ 99% aligned to the reference sequence [38].

The presence of antimicrobial resistance genes was determined by the Comprehensive Antibiotic Resistance Database (CARD) with the “perfect” and “strict” default settings for sequence analysis [41] and Task Template AMRFinderPlus 3.2.3 available in the Ridom SeqSphere + 7.0 software using the EXACT method: 100% sequence match over 100% of the length of a protein in the database that is not a named allele. The BLAST alignment is >90% of length and >90% identified to a protein in the AMRFinderPlus database.

### 2.10. Plasmid Detection

We used the PlasmidFinder 1.3 function to detect the plasmids [42], available from the Center for Genomic Epidemiology (http://www.genomicepidemiology.org/).

### 2.11. Profiling of CRISPR-Cas Loci

The search and characterization of the CRISPR matrices and the associated *cas* genes were determined with CRISPRCasFinder [43], which is available from the Institut de Biologie Intégrative de la Cellule and Université Paris-Saclay server (https://crisprcas.i2bc.paris-saclay.fr). The CRISPRDigger program was used to determine the type of CRISPR-Cas system [44].

## 3. Results and Discussion

Primary species identification by MALDI-TOF MS identified the six strains isolated from powdered milk in the Czech Republic between 2010 and 2014 as *C. sakazakii*. Further analysis by WGS identified four different STs among the six isolates (ST1: 13755-1B, 12683-1; ST145: 6227; ST245: 1847, 13755-1A; ST297: 12683-2A) (Table 1), and isolates with concordant STs shared the same O-serotype-specific gene loci for *gnd* and *galF* (ST1: 2 (*galF*) and 1 (*gnd)*; ST145: 25 and 23; ST245: 45 and 58; ST297: 17 and 18).

**Figure 1:**
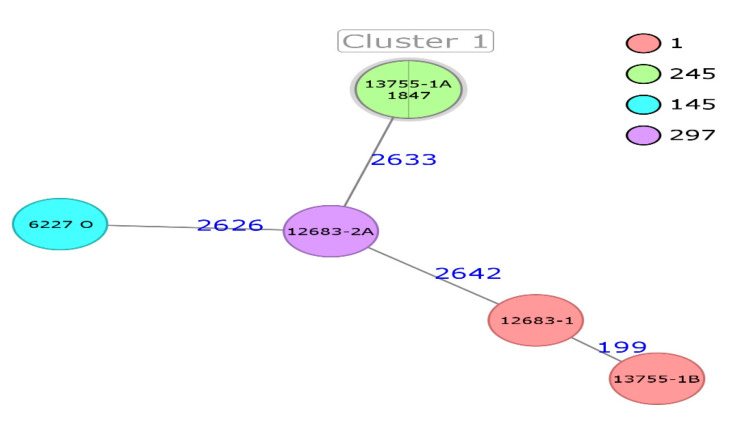
Minimum spanning tree (MST) of six *Cronobacter sakazakii* strains isolated from powdered milk (PM). The MST calculation was based on the defined cgMLST scheme comprising 2831 target genes. Isolates are represented as colored circles according to classical multilocus sequence typing (MLST). Blue numbers accord to the allelic difference between isolates. The isolates with closely related genotypes are marked as a cluster.

Calculation of a Minimum Spanning Tree (MST) (Figure 1) revealed four singleton isolates with a maximum allelic difference of 2642 and identified the presence of one cluster comprising two isolates, both belonging to ST245 and sharing the same set of cgMLST loci (isolates 13755-1A and 1847). To date, *C. sakazakii* ST245 has been primarily isolated from clinical cases; however, it has not been previously described in PIF. Meanwhile, *C. sakazakii* ST145 has been isolated in spices in China and soil in Germany (www.PubMLST.org/cronobacter/). Previously isolated from PIF, ST1 was detected twice in the present study and has been associated with fatal meningitis, septicemia, and urinary tract infections [45]. The detection of these *C. sakazakii* clones (ST1, ST245) in PIF and human samples confirms contaminated PIF as a possible source of infection and a potential health risk to infants. Lepuschitz et al. [38] concluded that the accurate identification of *C. sakazakii* is still a diagnostic challenge for many laboratories, and the current use of incorrect or outdated detection schemes would explain the low prevalence of *C. sakazakii* clinical isolates found in their EU study. Whole-genome sequencing can be applied to foodborne outbreaks to establish epidemiological links. It can also identify virulence loci, antibiotic resistance, and genotype, thus facilitating more accurate risk management [46].

**Figure 2:**
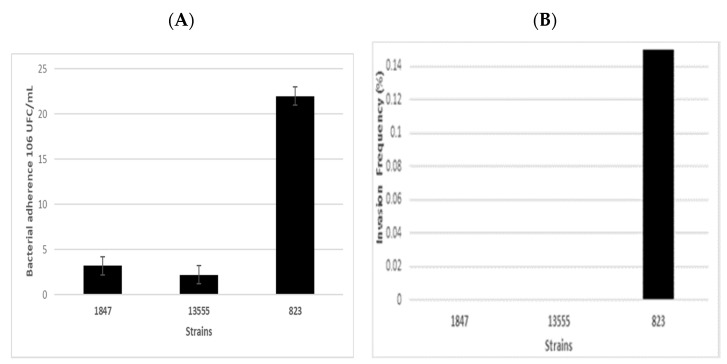
Bacterial adherence (**A**) and invasion frequency (**B**) of *Cronobacter sakazakii* on the neuroblastoma (NT) cell line.

*Cronobacter* spp. exhibit diverse virulence factors in the pathogenic process to intestinal cells such as adherence, invasion, toxin and hemolysin genes, and the ability to resist destruction by human serum [47,48,49,50]. Only strains 1847 (ST245) and 13755-1B (ST1) were selected to conduct the assays in a cell line because the STs were previously isolated from clinical cases. The strains were able to adhere to the cell line N1E-115 ATCC CRL-2263 with values of 2.2 and 3.2 × 10^6^ CFU/mL for strains 13755 and 1847, respectively (Figure 2). Adherence to intestinal cells is the first step in the pathogenic process [51]. This virulence characteristic has been studied in different cell lines such as N1E-115, HEp-2, CaCo-2, HBMEC, and IEC-6; the results have shown different variations depending on the type of cell line and strain being used [48,49,52]. The *C. sakazakii* strains isolated in clinical cases exhibited adherence rates that are higher (0.915%) than those from other sources (0.0002%) [23]. For the invasion assay, only strain 1847 was able to invade at a lower rate (0.00073%) compared with results reported by Mange et al. [48], Townsend et al. [49], Parra-Flores et al. [20], and Holý et al. [23].

Virulence factors must be studied to understand the pathogenic process and its relationship with the host [53]. In the present study, when evaluating the presence of six virulence factors by PCR, strain 13755-1B amplified three genes *ompA*, *aut*, and *fliC* and strain 1847 amplified the four genes *ompA*, *aut*, *fliC*, and *hlyA* (Table 2).

The results of the analysis of the whole genome of the six *C. sakazakii* strains was the presence of 28 virulence genes; these were grouped as flagellar or outer membrane proteins, chemotaxis, hemolysins, and invasion (*labp*), plasminogen activator (*cpa*), colonization (*mviN*), transcriptional regulator (*sdiA*), and survival in macrophages (Table 3). Virulence values determined by WGS were comparable to the PCR results. For example, the *hly* gene was amplified by PCR only for strain 1847, although it was present in both strains when analyzed in silico by WGS. Meanwhile, the *inv* gene was not detected by PCR in neither of the two evaluated strains; however, it was present in strain 1847 when analyzed by WGS. These results in strains 1847 and 13755-1B concur with the previously mentioned invasion and adherence assays. The *cpa* gene was not amplified by PCR and was not present in WGS. This can be explained by the fact that a greater number of strain activations for experimental work have produced some mutations that cause changes or non-expression of a certain phenotypic or genotypic quality in the strains being evaluated [54]. The OmpA protein is more than 80% the same as has the *E. coli* K1 protein, which significantly participates in the invasion of neonatal blood–brain barrier cells [55]. The proteins OmpA and OmpX of *Cronobacter* spp are important for adhesion to the CaCo-2 and INT-407 cell lines; they are also capable of causing damage to intestinal cells and eliminating villi [28,48]. The Cpa protein is considered as a key virulence factor involved in serum resistance and invading *C. sakazakii*. Evolutionary evidence suggests that the *cpa* locus can be a specific locus for *C. sakazakii* and *C. universalis* [26]. However, some ST8 clinical strains of *C. sakazakii* that have the pESA3 virulence plasmid do not have *cpa* yet remain are considered extremely virulent. This suggests there are other virulence factors besides *cpa* that can be responsible for the infection [56]. The Hly (type III hemolysin) is a protein of the external membrane found in several pathogens with hemolytic ability [57,58]. It was present when analyzing the *C. sakazakii* BAA-894 strain isolated from the 2001 NICU outbreak in the United States [59]. The autotransporter gene (*aut*) has been identified in *E. coli* and other Enterobacteriaceae; it is associated with adherence, aggregation, invasion, biofilm formation, and toxicity [60]. The *fliC* gene is a flagellar protein and a subunit of the flagellar organelle; it is primarily responsible for bacterial motility, adherence ability, and virulence traits of microorganisms [61]. Hoeflinger and Miller [62] evaluated flagella-mediated autoaggregation and determined that the flagella play an important role in the pathogenesis of *C. sakazakii*. Aldubyan et al. [63] established the significant role of flagella in the adherence and invasion of pathogens that contain *fliC*. Decreased motility has been related to the loss of the *fliC* gene. Dingle et al. [64] determined the important role of the FliC and FliD flagellar subunit, which increased the adherence to Caco-2 cells compared with the wild type; mutants are also more virulent. These virulence factors established as flagella-associated genes, outer membrane protein genes, and some regulators can be related to motility, biofilm formation, and virulence characterization in *Cronobacter* [65].

When evaluating the antibiotic resistance profile of the two selected strains (1847 and 13755-1B), both were resistant to cephalothin, whereas strain 13755-1B was resistant to ceftazidime and strain 1847 to ampicillin. Several authors have identified the resistance of *C. sakazakii* to cephalothin, ceftazidime, and ampicillin [18,66,67]. Some strains were resistant to ampicillin, cephalothin, and cefotaxime in a collection of *Cronobacter* spp. strains isolated by Parra et al. [68]. In contrast, Holý et al. [23] found no resistant strains. A recent study by Parra et al. [17] found that 100% of the *C. sakazakii* strains isolated from powdered milk were resistant to cefotaxime and ampicillin. In addition, resistance to cefepime and amikacin was 60% and 40% for ceftriaxone. In addition, one strain was resistant to six of the 12 evaluated antibiotics (54.5%), while another *C. sakazakii* isolated strain was resistant to five (50%). Resistance values in the present study are higher than those reported in the current literature, and should be studied in the event of the appearance of multi-resistant *C. sakazakii* strains in powdered milk (PM) and the associated health risk to infants and children.

A total of 12 genes were detected in silico by CARD, thus conferring antibiotic resistance against beta-lactams, fluoroquinolones, aminoglycosides, and phosphonates (Table 4). Of the 12 antibiotic resistance genes, including antibiotic efflux (n = 8), antibiotic target alteration (3), and antibiotic target protection (1), 11 were present in all the isolates. The antibiotic target protection gene (*vgaC*) was present in only three of the six isolates and the *marA* gene, whose function is as a transcription factor that upregulates multidrug efflux and downregulates membrane permeability, was found in all the isolates. As in our study, Aly et al. [69] found *msbA*, *emrR*, *H-NS*, *emrB*, *marA*, *CRP*, and *PBP3* to be associated with resistance to several antibiotics such as beta-lactams, tetracycline, macrolide, fluoroquinolone, penams, cephalosporin, and cephamycin. Active flow pumps provide a mechanism to increase resistance by improving the survival of enterobacteria in the gastrointestinal tract of the host and allowing the invasion of microvascular endothelial cells in the brain [70,71]. Studies have demonstrated that the abuse of antibiotics in food environments and the presence of several antibiotic resistant operons (*mar*) can cause *Cronobacter* spp. to develop resistance to many different antibiotics [66,67,72].

When complementing our research with the AMRFinderPlus tool, strains 1847, 13755-1A, 12683-1, and 12683-2A exhibited the *mcr-9.1* gene associated with resistance to colistin and the gene *bla_CSA_* that provides resistance to cephalothin in 100% (6/6) of the strains evaluated in the study. The *mcr-9.1* gene is considered as a new gene that can attribute phenotypic resistance to colistin in various *Enterobacteriaceae* spp. reported in *Salmonella typhimurium*, *Escherichia coli,* and *Enterobacter hormaechei* in 2019. They can circulate without being detected, unless induced by colistin [73,74,75]. The presence of mobile genes that are resistant to colistin (*mcr*) is a worldwide concern because colistin is perceived as a last resort antimicrobial to treat infections caused by multi-resistant *Enterobacteriaceae* [76]. The *bla_CSA_* family of genes, resistant to class C β-lactamase, was described by Müller et al. [77]. The members of this family of β-lactamases are not inducible and are regarded as cephalosporinases. Jang et al. [56] found that the *C. sakazakii* strains isolated from houseflies had class C *bla_CSA_* resistance genes. They also encountered variants of class C *bla* resistance genes identified as *CSA-2* or *CSA-1.*

Strains 1847 and 13755-1A, present in IncFII (pECLA) plasmid (Table 5), are associated with antibiotic resistance genes in *Cronobacter*, such as carbapenemase genes ∆intI1–*bla_IMP-26_*, *bla_CSA_*, *bla_CMA_*, and IS26 flanking *bla_SFO-1_* [77,78].

Strain 12683-2A exhibited an IncFIB (pCTU1) plasmid, which is also associated with antibiotic resistance (Table 4). Meanwhile, pCTU1 encodes similar gene groups or gene clusters comprising the plasmid backbone, a replication gene similar to RepFIB (*repA*), two iron acquisition systems, a siderophore similar to aerobactin (called cronobactin), a group of iron ABC transport genes, and several species-specific virulence gene determinants such as the *cpa* gene [79]. Therefore, the absence of pCTU1 type plasmids in strains 1847 and 13755-1A can explain the non-detection of the *cpa* gene by PCR and WGS [56].

The CRISPR-Cas systems are associated with the acquisition of mobile genetic material through horizontal transfer; they are regarded as an immunity system. These contain information that the bacteria have acquired through the virus and plasmids. They consist of two principal components: a guide RNA (gRNA) and a non-specific endonuclease associated with genes that code for *cas*, both of which are indispensable for the activity and incorporation of genetic material [80]. When analyzing the genomes of the six *C. sakazakii* strains in the present study, we found that 100% (6/6) exhibited a series of repeated sequences and spacers, which constitute matrices associated with type I-F and I-E CRISPR systems; the four strains 13755-1A, 13755-1B, 12683-2A, and 12683-1 were included in both systems (Table 6). Regarding the characteristics of the CRISPR matrices, we encountered five different repeated consensus sequences associated with the type I-E system, and the sequence GTGTTCCCCGCGCGAGCGGGGATAAACCG was the most frequent because it was associated with four of the six strains under study; however, strains 13755-1A and 6227 exhibited only one repeated consensus sequence. For the type I-F system, three different repeated sequences were found; the sequence GTTCACTGCCGTACAGGCAGCTTAGAAA was the most frequent and sequence TTTCTAAGCTGCCTGTACGGCAGTGAAC was distinctive of strain 12683-A. Although the sequences that characterize each system can be identical, one aspect that differs from strain to strain is the number of repeated sequences and spacers that characterize them. The above-mentioned strain had the largest matrices, with a maximum of up to 49 repeated sequences and 53 spacers for the I-F system and 58 repeated sequences and 62 spacers for the I-E system. This provides us with knowledge about the information this microorganism is acquiring from the bacteriophages and plasmids.

The strains that exhibit the same sequence type can have different CRISPR matrices; for example, strains 1847 and 13755-1A associated with ST245 exhibited different types of CRISPR-Cas systems, whereas the opposite was observed in strains 12683-1 and 13755-1B, which had the same sequence type and the same CRISPR matrices. This is relevant because this system has been used as a typing method in different microorganisms. When analyzing 29 genomes of *C. sakazakii* ST1, Ogrodzki and Forsythe [81] found that all the strains exhibited the same type I-E system and three spacer matrices with conserved patterns. In addition, the use of CRISPR spacer matrix profiles compared with MLST show greater intra-species discrimination power; it is a useful tool to study future *Cronobacter* outbreaks by more accurately relating clinical cases, food sources, and production sites associated with and outbreak [82]. While it was initially believed that *C. sakazakii* had only one type of CRISPR system, Zeng et al. [83] indicated that 94.5% of the analyzed *C. sakazakii* strains showed more than one CRISPR and these were in conserved zones of its genome. Although each type of CRISPR-Cas system is characterized by certain associated *cas* genes, the *cas1* and *cas2* genes are indispensable for integrating and processing the information acquired by the bacterium. It has also been suggested that if any of these genes are not present, the system loses the ability to acquire information, and therefore can no longer integrate information into the CRISPR locus [84].

## 4. Conclusions

*Cronobacter sakazakii* was confirmed in six of the 1450 evaluated powdered milk samples (PM). The *C. sakazakii* isolates exhibited virulence factors, resistance genes to beta-lactam antibiotics, and a colistin (*mrc 9.1*) strain, which represent risk for infants consuming this product. Combined molecular methodologies based on WGS improve *C. sakazakii* identification and provide more reliable information for decision-making by health authorities to protect the infant population that consumes PM.

## Figures and Tables

**Table 1:** *Cronobacter sakazakii* strains isolated from powdered milk.

Strain	NGS ID	Identification	ST	Source	Country	Year of Isolation	Manufacturer
* 1847	510284-18	*C. sakazakii*	245	Powdered milk	Czech Republic	2010	A
13755-1A	510192-19	*C. sakazakii*	245	Powdered milk	Czech Republic	2014	B
13755-1B	510193-19	*C. sakazakii*	1	Powdered milk	Czech Republic	2014	B
12683-1	510285-19	*C. sakazakii*	1	Powdered milk	Czech Republic	2014	B
12683-2A	510194-19	*C. sakazakii*	297	Powdered milk	Czech Republic	2014	B
6227	510555-19	*C. sakazakii*	145	Powdered milk	Czech Republic	2014	C

* ID 1482: PubMLST/*Cronobacter*. NGS ID: Next Generation Sequencing Identification; ST: sequence type.

**Table 2:** Presence of virulence factors by polymerase chain reaction (PCR).

*C. sakazakii* Strain (ST)			Gene			
	*hlyA*	*ompA*	*aut*	*fliC*	*inv*	*cpa*
13755-1B (1)	−	+	+	+	−	−
1847 (245)	+	+	+	+	−	−
823 (ATCC BAA-894) (1) control strain	+	+	+	+	+	+

The presence of a gene is represented by “+” and the absence of a gene is represented by “−”.

**Table 3:** Virulence gene distribution among six strains of *Cronobacter sakazakii* by whole-genome sequencing (WGS).

Virulence Gene	Function	1847 (ST245)	13755-1A (ST245)	13755-1B (ST1)	12683-1 (ST1)	12683-2A (ST 297)	6227 (ST145)
*flgB*	motility	+	+	+	+	+	+
*flgK*	flagellar hook-associated protein 1	+	+	+	+	+	+
*flgL*	flagellar hook-associated protein 3	+	+	+	+	+	+
*flgM*	negative regulator of flagellin synthesis	+	+	+	+	+	+
*flgN*	flagella synthesis FlgN protein	+	+	+	+	+	+
*flhD*	flagellar hook-associated protein 2	+	+	+	+	+	+
*fliA*	flagellar operon FliA	+	+	+	+	+	+
*fliC*	flagellin	+	+	+	+	+	+
*fliD*	flagellar hook-associated protein 2	+	+	+	+	+	+
*fliR*	flagellar biosynthetic FliR protein	+	+	+	+	+	+
*fliT*	flagella FliT protein	+	+	+	+	+	+
*fliZ*	FliZ protein	+	+	+	+	+	+
*lolA*	outer membrane lipoprotein carrier protein	+	+	+	+	+	+
*motB*	chemotaxis MotA protein	+	+	−	+	+	+
*ompW*	transmembrane transport	+	+	+	+	+	+
*sdiA*	LuxR family transcriptional regulator	+	+	+	+	+	+
*slyB*	outer membrane lipoprotein SlyB	+	+	−	+	+	+
*tolC*	outer membrane channel protein	+	+	+	+	+	+
*MsgA*	survival in macrophage	+	+	+	+	+	+
*MviN*	protective immunity and colonization in Salmonella	+	+	+	+	+	+
*cpa*	plasminogen activator	−	−	+	+	+	+
*hha*	hemolysin expression modulating protein	+	+	+	+	+	+
*hly III*	hemolysin III	+	+	+	+	+	+
*ompA*	adhesion cell; cell death induction; biofilm formation	+	+	+	+	+	+
*ompX*	adhesion cell	+	+	+	+	+	+
*blc*	outer membrane lipoprotein	+	+	+	+	+	+
*cheR*	chemotaxis protein methyltransferase	+	+	−	+	+	+
*cheY*	response regulator of chemotaxis family	+	+	+	+	+	+
*labp*	epithelial cell invasion and lipid A production by LpxA	+	−	+	−	−	−

ST: Sequence type. The presence of a gene is represented by “+” and the absence of a gene is represented by “−”.

**Table 4:** Antibiotic-resistant genes identified by Comprehensive Antibiotic Resistance Database (CARD).

Best Hit Antibiotic Resistance Ontology (ARO)	Drug Class	Resistance Mechanism	1847 (ST245)	13755-1A (ST245)	13755-1B (ST1)	12683-1 (ST1)	12683-2A (ST297)	6227 (ST145)
*CRP*	fluoroquinolone antibiotic; macrolide antibiotic; penam	antibiotic efflux	+	+	+	+	+	+
*marR*	monobactam; triclosan; rifamycin antibiotic; penem; cephamycin; fluoroquinolone antibiotic; penam; phenicol antibiotic; glycylcycline; tetracycline antibiotic; cephalosporin; carbapenem	antibiotic efflux; reduced antibiotic permeability	+	+	+	+	+	+
*H-NS*	fluoroquinolone antibiotic; macrolide antibiotic; penam; tetracycline antibiotic; cephalosporin; cephamycin	antibiotic efflux	+	+	+	+	+	+
*EF-Tu*	elfamycin antibiotic	antibiotic target alteration	+	+	+	+	+	+
*marA*	fluoroquinolone antibiotic; triclosan; rifamycin antibiotic; penam; phenicol antibiotic; glycylcycline; tetracycline antibiotic; cephalosporin	antibiotic target alteration; antibiotic efflux	+	+	+	+	+	+
*mrB*	fluoroquinolone antibiotic	antibiotic efflux	+	+	+	+	+	+
*emrR*	fluoroquinolone antibiotic	antibiotic efflux	+	+	+	+	+	+
*adeF*	tetracycline antibiotic; fluoroquinolone antibiotic	antibiotic efflux	+	+	+	+	+	+
*msbA*	nitroimidazole antibiotic	antibiotic efflux	+	+	+	+	+	+
*vgaC*	streptogramin antibiotic; pleuromutilin antibiotic	antibiotic target protection	+	+	−	−	+	−
*GlpT*	fosfomycin	antibiotic target alteration	+	+	+	+	+	+
*PBP3*	penam; cephalosporin; cephamycin; monobactam; carbapenem	antibiotic target alteration	+	+	+	+	+	+

The presence of a gene is represented by “+” and the absence of a gene is represented by “−”.

**Table 5:** Presence of plasmids in *Cronobacter sakazakii* strains by whole genome sequencing (WGS).

Strains (ST)	Plasmids	Accession Number	Function
1847 (1); 13755-1A (245)	IncFII(pECLA)	CP001919	Antibiotic resistance
12683-2A (297)	IncFIB(pCTU3)	FN543096	

**Table 6:** Profiling of CRISPR-Cas loci among *Cronobacter sakazakii* strains.

Strains	Sequence Type (ST)	Operon Structure Type	Number of CRISPR Arrays per Strain	Maximum Number of Spacers per Strain	Sequences with Cas Cluster	Repeat Consensus/*cas* Genes
1847	245	Type I-E Cas	29/7	32/9	1	GTTCACTGCCGTACAGGCAGCTTAGAAA/CTGTTCCCCGCGCGAGCGGGGATAAACCG/ Cas3_0_I, Cse1_0_IE, Cse2_0_IE, Cas7_0_IE, Cas5_0_IE, Cas6_0_IE, Cas1_0_IE, Cas2_0_IE.
13755-1A	245	Type I-F Cas Type I-E Cas	10 18	9 17	1 1	GTTCACTGCCGTACAGGCAGCTTAGAAA/ Cas1_0_IF, Cas3-Cas2_0_IF, Cas6_0_IF, Csy1_0_IF, Csy2_0_IF, Csy3_0_IF. CGGTTTATCCCCGCTCGCGCGGGGAACAC/ Cas3_0_I, Cse1_0_IE, Cse2_0_IE, Cas7_0_IE, Cas5_0_IE, Cas6_0_IE, Cas1_0_IE, Cas2_0_IE.
13755-1B	1	Type I-E Cas Type I-F Cas	22/30 14	24/32 16	1 1	CTGTTCCCCGCGCGAGCGGGGATAAACCG/GTGTTCCCCGCGCGAGCGGGGATAAACCG/ Cas3_0_I, Cse1_0_IE, Cse2_0_IE, Cas7_0_IE, Cas5_0_IE, Cas6_0_IE, Cas1_0_IE, Cas2_0_IE. GTTCACTGCCGTACAGGCAGCTTAGAAA/ Cas1_0_IF, Cas3-Cas2_0_IF, Cas6_0_IF, Csy1_0_IF, Csy2_0_IF, Csy3_0_IF.
12683-2A	297	Type I-F Cas Type I-E Cas	49 20/58	53 22/62	1 1	TTTCTAAGCTGCCTGTACGGCAGTGAAC/ Cas1_0_IF, Cas3-Cas2_0_IF, Cas6_0_IF, Csy1_0_IF, Csy2_0_IF, Csy3_0_IF. CTGTTCCCCGCGCGAGCGGGGATAAACCG/GTGTTCCCCGCGCGAGCGGGGATAAACCG/ Cas3_0_I, Cse1_0_IE, Cse2_0_IE, Cas7_0_IE, Cas5_0_IE, Cas6_0_IE, Cas1_0_IE, Cas2_0_IE.
12683-1	1	Type I-E Cas Type I-F Cas	28/31 14	29/32 15	1 1	CTGTTCCCCGCGCGAGCGGGGATAAACCG/GTGTTCCCCGCGCGAGCGGGGATAAACCG/ Cas3_0_I, Cse1_0_IE, Cse2_0_IE, Cas7_0_IE, Cas5_0_IE, Cas6_0_IE, Cas1_0_IE, Cas2_0_IE. GTTCACTGCCGTACAGGCAGCTTAGAAA/ Cas1_0_IF, Cas3-Cas2_0_IF, Cas6_0_IF, Csy1_0_IF, Csy2_0_IF, Csy3_0_IF.
6227	145	Type I-E Cas	15/13	16/12	1	CGGTTTATCCCCGCTCGCGCGGGGAACGG/GTGTTCCCCGCGCGAGCGGGGATAAACCG// Cas1_0_IE, Cas2_0_IE, Cas5_0_IE, Cas6_0_IE, Cas7_0_IE, Cse1_0_IE, Cse2_0_IE.
